# Reduction of hexavalent chromium using bacterial isolates and a microbial community enriched from tannery effluent

**DOI:** 10.1038/s41598-022-24797-z

**Published:** 2022-11-23

**Authors:** Eva Plestenjak, Barbara Kraigher, Simona Leskovec, Ines Mandic Mulec, Stefan Marković, Janez Ščančar, Radmila Milačič

**Affiliations:** 1 Department of Microbiology, Biotechnical Faculty, University of Ljubljana, Večna Pot 111, 1000 Ljubljana, Slovenia; 2 Department of Environmental Sciences, Jožef Stefan Institute, Jamova 39, 1000 Ljubljana, Slovenia; 3 Jožef Stefan International Postgraduate School, Jamova 39, 1000 Ljubljana, Slovenia

**Keywords:** Microbiology, Environmental sciences

## Abstract

We investigated microbial growth in increasing concentrations of hexavalent chromium (Cr(VI)) and its reduction by a microbial community enriched from tannery effluent and by the bacterial strains isolated from the enriched community. The bacterial growth was monitored by measuring the optical cell density (OD_650_), while the Cr(VI) concentration in the samples was determined using spectrophotometry and liquid chromatography hyphenated to inductively coupled plasma mass spectrometry (HPLC–ICP–MS). At a Cr(VI) concentration of 100 mg/L, the isolates affiliated with *Pseudomonas aeruginosa* (*P. aeruginosa*) reached higher optical cell densities, but were in general less effective for Cr(VI) reduction than the isolates affiliated with *Mammaliicoccus sciuri* (*M. sciuri*). All three *M. sciuri* isolates and only one of the seven *P. aeruginosa* isolates were able to reduce 50% of the Cr(VI) with an initial concentration of 100 mg/L within 24 h (pH 7.1), while the six isolates affiliated with *P. aeruginosa* were less effective. Compared to the isolated, individual bacterial strains, the enriched microbial community was better adapted to the elevated Cr(VI) concentrations, but needed a longer time (48 h) to reduce the Cr(VI) with the same efficacy as the most efficient individual isolates. The ability of the enriched microbial community and the isolated bacterial strains to reduce the Cr(VI) highlights their potential for use in the rapid bioremediation of wastewaters contaminated with Cr(VI).

## Introduction

The toxicity of chromium (Cr) depends a great deal on its chemical form. Hexavalent Cr (Cr(VI)) compounds are extremely toxic, exhibiting mutagenic and carcinogenic effects on living organisms^1^. In addition, soluble chromates are highly mobile in terrestrial and aquatic environments. The trivalent Cr (Cr(III)) species are far less toxic than Cr(VI). In the environment, at a pH higher than 6, Cr(III) forms relatively insoluble compounds, mainly oxides and hydroxides^2^. Cr(VI) is more stable in the alkaline pH ranges, while in acidic conditions it is rapidly reduced by naturally occurring reducing agents like Fe(II), sulfides, and organic matter. Cr(VI) can also be reduced by microorganisms. After reduction, it precipitates and/or adsorbs in the form of Cr(III) species^3^. Metallic Cr and its salts are widely used in the steel industry^4^, electroplating^5^ for tanning leather and treating wood^6,7^, producing Cr pigments^8^, the automotive industry^9^, and producing glass and ceramics^10^. Cr is also present in cement and cement products^11^. Because of its widespread use, large amounts of Cr have been released into the environment^12^. Environmental burden may represent also Cr-rich waste materials generated in a variety of industrial processes^13^. If Cr is released into the environment in its hexavalent form in untreated industrial effluents^14^ or as a result of accidental spills^15^, it contaminates soil, reaches watercourses, enters groundwater and contaminates drinking water. The presence of higher concentrations of Cr(VI) in wastewaters can also inhibit the sludge-nitrification process in treatment plants^16^. In order to prevent or minimize the toxic effects of Cr(VI) on the environment and living beings, it is necessary to remove the Cr(VI) or reduce it to the far less toxic Cr(III). The remediation strategies involve physical methods such as adsorption, ion-exchange and electrodialysis, and chemical reduction by Fe(II) sulfate, sulfides, sulfites and sulfur dioxide^17^. Recently, palladium-based nanoparticles characterized by XPS, XRD, p-XRD, TEM, TEM–EDX and HR-TEM were used for the catalytic reduction of Cr(VI)^18,19^. A variety of other nanomaterials have also been applied to remove Cr(VI) from contaminated waters^20^ and soils^21^. A promising remediation strategy is the use of living organisms, including bacteria, fungi, yeast, algae, and plants, among which bacteria and fungi have shown the highest remediation capacity^17,22^. The bioremediation of Cr(VI) includes the mechanisms of biosorption on the surface of dead or live biomass, bioaccumulation within the cell wall, and biotransformation, a process in which Cr(VI) is reduced to Cr(III). Biosorption involves the formation of a chemical bond between the Cr(VI) and functional groups (e.g., proteins, glycoproteins, polysaccharides, glycolipids) present on the cell walls. Upon adsorption, the Cr(VI) either precipitates on the surface of the microbial cell or is reduced to Cr(III) (extracellular reduction). Soft X-ray spectromicroscopy was used to study these processes^23^. In prokaryotes or eukaryotes, oxyanion chromate is actively transported across biological membranes through the sulfate transporters. Inside the cell, it is rapidly reduced to Cr(III) (intracellular reduction) via the formation of unstable intermediates of pentavalent and tetravalent Cr species^24^. In Cr-resistant bacteria, different chromate reductases catalyze the reduction of Cr(VI) to Cr(III), mediating the transfer of electrons from electron donors to the Cr(VI)^25,26^. Both aerobic and anaerobic microorganisms can reduce Cr(VI). The bacterial remediation of Cr(VI) using native, non-pathogenic bacterial strains is a fast, safe, and economically viable process^27^. Gram-negative or gram-positive bacteria can effectively remove Cr(VI) through biosorption, biotransformation or by involving both mechanisms^17,28,29^. Gang et al.^24^ studied the adaptation mechanisms of microorganisms to long-term Cr(VI) stress at the proteome level. They found that an increasing concentration of Cr(VI) in the culture media can significantly improve the resistance capacity of microorganisms and their adaptation to elevated Cr(VI) concentrations, thus enabling the reduction of Cr(VI) and effective bioremediation. The resistance capacity to Cr(VI) is stimulated by cellular motility, efflux, antioxidant activity, protection against oxidative stress by detoxifying enzymes, and DNA repair systems^24,26^.

A wide range of Cr-resistant bacteria such as *Bacillus* species^30^, *Lactobacillus* strains^27^, *Bacillus amyloliquefaciens*^31,32^, *Bacillus cereus*^33^, *Bacillus methylotrophicus* strain^25^ *Stenotrophomonas maltophilia*^26^, *Staphylococcus sciuri*^34^ as well as *Cellulosimicrobium funkei*^35^ and a mixed bacterial consortium^36^ have been isolated from chromium-contaminated soils^31,32,36^, tannery effluents^26,30,33–35,37^ and tannery sludge^25^.

In Slovenia there are several disposals of tannery waste from abandoned leather industries, rich in Cr(III), which was used for leather tanning. Since chromate-reducing microorganisms have been identified as a potential candidate for the bioremediation of Cr(VI)-contaminated sites, the objectives of this study were: (i) to enrich the microbial community from tannery effluent that can grow at elevated Cr(VI) concentrations, (ii) to isolate Cr(VI)-resistant bacterial strains, and (iii) to monitor the reduction capacity of isolated bacterial strains and the microbial community for reducing Cr(VI) in aqueous samples using chemical speciation methods.

## Materials and methods

### Tannery-effluent sample collection and characterization

A sample of effluent from the landfill of a former tannery in Vrhnika, Slovenia, which operated until 2008, was collected in April 2018 in a sterile, 50 mL plastic tube and stored at 4 °C.

To determine the number of colony forming units (CFU), the effluent sample was appropriately diluted in a physiological solution, inoculated into an R2A agar medium (Sigma Aldrich, Burlington, MA, USA), and colonies were counted after incubation at 28 °C for 24 h. To determine the number of sporulating bacteria the sample was incubated at 90 °C for 20 min. A total of 100 µL of the sample was then inoculated on the LB agar medium (Sigma Aldrich) and colonies were counted after incubation at 37 °C for 24 h.

A DR 3900 Hach Lange (Düsseldorf, Germany) spectrophotometer was used for the determination of the total organic carbon (TOC) using the reagent-kit method (cuvette test LCK381). The TOC in the LB medium was calculated as the difference between the total carbon (TC) and total inorganic carbon (TIC).

### Enrichment cultures

To adapt the microorganisms to the elevated Cr(VI) concentrations, the microbial community from the tannery effluent was enriched in the LB medium with increasing Cr(VI) concentrations. First, the wastewater sample was added to the LB medium treated with 34.5 mg/L Cr(VI) and incubated at 37 °C and 200 rpm until the medium changed color from orange to grey-green. The color change of the LB medium was observed after 24 h of incubation, and a pre-adapted culture to a concentration of 35.4 mg/L Cr(VI) was added to a fresh LB medium with a final concentration of 106 mg/L Cr(VI) and incubated at 37 °C and 200 rpm for 3 days. Following the same procedure, the medium was enriched with 177 and 248 mg/L Cr(VI), and the samples were incubated for 4 days. The next enrichments were followed by additions of Cr(VI) with final concentrations of 354, 424, 459, and 600 mg/L, while the samples were incubated for 9, 11, 12, and 14 days, respectively.

Prior to the inoculation of the enrichment culture from the LB medium with lower concentration to the LB medium with the higher Cr(VI) concentration, an aliquot of the culture was diluted appropriately in a physiological solution, inoculated onto the LB agar plate treated with the same Cr(VI) concentration as in the enrichment culture and the plates were incubated at 37 °C.

### Isolation and characterization of bacteria capable of growing at high concentrations of Cr

Isolated strains were obtained from the LB agar plates treated with 106 or 177 mg/L Cr(VI). From the LB medium treated with 106 mg/L Cr(VI), a diluted culture was inoculated onto the LB agar plate with a Cr(VI) concentration of 106 mg/L to obtain isolates 3001, 3002, and 3003. Inoculation of the diluted enrichment cultures treated with 106 mg/L Cr(VI) on LB agar plates with a Cr(VI) concentration of 177 mg/L gave isolates 3004, 3005, 3006, and 3007. Isolates 5008, 5009 and 50010 were obtained from the LB medium treated with 177 mg/L Cr(VI) and inoculated on LB agar plates with 177 mg/L Cr(VI). Isolates were selected based on morphological differences in color, shape, and colony edge and stored in 12% glycerol at − 80 °C.

#### Gram staining

Isolates were inoculated onto LB agar plates and incubated overnight at 37 °C. Gram staining was then performed with crystal violet dye and lugol, decolorized (15 s) with a drop of a mixture of acetone and ethanol (1:1) and then colorized with safranin. The shape and color of the bacteria in the stained preparations were observed with a microscope.

#### Molecular identification of the isolates

The isolated bacteria were grown in the LB medium overnight at 37 °C and 220 rpm. Overnight cultures of the Gram-negative strains 3001, 3002, 3003, 3004, 3006, 3007, and 5008 were then boiled for 10 min, and 6 µL of the boiled cells were used as a template for the amplification of 16S rRNA using the polymerase chain reaction (PCR). From strains 3005, 5009, and 50010, DNA was isolated using a chromosomal DNA-isolation kit. In this case a GenElute Bacterial Genomic DNA Kit (Sigma-Aldrich).

The 16S rRNA gene was amplified using universal primers 27 F (5′-AGAGTTTGATCCTGGCTCAG-3′) and 1406 R (5′-GACGGGCGGTGTGTRCA-3′). PCR reactions were performed in a final volume of 50 µL, comprising of 6 µL of DNA, 26.6 µL of Milli-Q water, 10 µL of PCR buffer 5 ×, 2 mM MgCl_2_, 200 mM dNTP, 0.2 mM of each primer 27 F and 1406 R and 1 U of Taq polymerase (Promega, Madison, WI, USA). The PCR was performed in a Biometra-UNO Thermoblock (Biotron, Göttingen) with the following thermocycling conditions: 5 min of initial denaturation at 95 °C, followed by 25 cycles of 30 s denaturation at 95 °C, 30 s of annealing at 53 °C and 2 min of extension at 72 °C; cycling was completed by a final extension step of 10 min at 72 °C. The PCR products were purified using a PCR purification kit from Invitrogen (Thermo Fisher Scientific) and sent to Macrogen Inc. for Sanger sequencing with the 27F and 1406R primers. The obtained sequences were manually proofread and assembled using MEGA11 software when the sequences obtained from both primers were of good quality^38^. The obtained partial 16S rRNA gene sequences (at least 1000 bp long) were compared with the available database nucleotide sequences using the Basic Local Alignment Search Tool (BLASTN, https://blast.ncbi.nlm.nih.gov/Blast.cgi) for an initial phylogenetic assignment and deposited in the GenBank database under accession numbers ON409639-ON409641 and ON430687-ON430693.

### Evaluation of Cr(VI) toxicity and its reduction

#### Growth of bacterial isolates at various concentrations of Cr(VI) and its reduction effectiveness

To evaluate the toxicity of Cr(VI) the bacterial growth was monitored for isolates treated with different Cr(VI) concentrations. The isolates were inoculated into an LB medium and incubated overnight at 37 °C and 200 rpm. A total of 50 µL of the overnight-incubated culture was then inoculated into 5 mL of LB medium treated with 0, 100, 200, 500, 1000, 1500, 2000, 2500, and 3000 mg/L Cr(VI). The optical density was determined using a Thermo Electron microplate reader (Thermo Fisher, Waltham, MA, USA) immediately after the inoculation (time zero) and 24 h after inoculation at 650 nm (OD_650_). The bacterial growth in 24 h was calculated by subtracting the OD_650_ at time zero from the OD_650_ at 24 h after inoculation.

To evaluate the effectiveness of each isolated strain in reducing the Cr(VI), the concentration of Cr(VI) was also determined after 24 h in the samples of bacterial culture grown in the LB media treated with 100 or 200 mg/L Cr(VI). To stop the bacterial activity, bacteria were removed from the LB media by filtration through 0.45 µm filters (Minisart filters, Sartorius Stedim Biotech GmbH, Goettingen, Germany), followed by micro-ultracentrifugation (5 min, 10,000 rpm) using Amnicon (Beverly, MA, USA) Ultra-4 (4 mL) Centrifugal Filter Devices tubes, Ultracel 3 K (cut-off 3000 Da) to remove all the cells. The bacterial-free centrifugate was stored at − 20 °C prior to the Cr(VI) analysis.

#### Growth of the enriched microbial community at various concentrations of Cr(VI) and its effectiveness for reduction

An enriched microbial community treated with 600 mg/L Cr(VI) was used to evaluate the toxicity of the Cr(VI). To prepare the inoculum, the microbial community was centrifuged (10 min, 11,000 rpm, 4 °C), and the cells were re-suspended in a physiological solution and centrifuged (10 min, 11,000 rpm, 4 °C). The supernatant was discarded, and the cells were re-suspended in a sterile LB medium. A total of 3 mL of the inoculum, thus prepared, was added to 120 mL of LB medium treated with 0, 5, 25, 50, 100, 250, or 500 mg/L Cr(VI), and the samples were incubated at 37 °C and 200 rpm for up to 48 h. The same experiment was performed without adding the inoculum to media treated with Cr(VI). In each sample, OD_650_ was determined using a MA 9510 spectrophotometer (Mettler Toledo, Ljubljana, Slovenia) to examine the bacterial growth at time zero, 24 h, and 48 h after incubation. The number of cells was then determined by counting the colony forming units (CFU/mL) on the LB agar plates.

To determine the total Cr and Cr(VI), a sample aliquot was filtered through a 0.45-μm filter and then micro-ultracentrifuged (10 min, 8000 rpm, 4 °C). The bacteria-free centrifugate was stored at − 20 °C prior to analysis.

### Determination of total Cr, Cr(VI) and pH in tannery effluent, microbial community and culture isolates samples

The total Cr was determined by ICP-MS on an Agilent 7900 instrument (Tokyo, Japan). The tannery effluent sample was digested in *aqua regia* according to the procedure in ISO 15587^39^.

The Cr(VI) in the tannery effluent was determined with the HPLC–ICP–MS, using a strong anion-exchange Mono Q HR 5/5 (GE Healthcare Bio-Sciences, Uppsala, Sweden) column^40^. Chromatographic separations were performed on an Agilent series 1200 quaternary pump equipped with a sample injection valve, Rheodyne, model 7725i (Cotati, Ca, USA) fitted with a 0.2 mL injection loop. The data were treated with Agilent MassHunter software. The data processing was based on the peak area. The ICP-MS operating parameters are summarized in Table S1 (Supplementary material).

The Cr(VI) in the microbial community and the culture isolates was determined by spectrophotometry on a DR 3900 Hach Lange Spectrophotometer using the reagent kit method (cuvette tests LCK313) for a rapid determination of the chromate ion, according to the manufacturer’s instructions, using 2 mL of sample*.* Spectrophotometry is based on the rapid reaction of Cr(VI) with 1,5 diphenylcarbazide in an acidic solution. In this reaction, simultaneous oxidation of the reagent to diphenylcarbazone and Cr(VI) reduction to Cr(III) occurs. The newly formed Cr(III) reacts rapidly with the diphenylcarbazone, forming a magenta-colored Cr(III)-diphenylcarbazone complex, which is monitored at 540 nm. If 1,5 diphenylcarbazide is added directly to the Cr(III) solution, the color develops extremely slowly. This enables an accurate determination of the Cr(VI) in the presence of Cr(III)^41^. All the solutions used in the Cr and Cr(VI) determinations were made from ultrapure 18.2 MΩ cm water (Milli-Q) obtained from a Direct-Q 5 Ultrapure water system (Millipore Watertown, MA, USA). Sodium chloride (NaCl) (s.p.) (Merck, Darmstadt, Germany) was used in the HPLC separations. Stock standard solutions of Cr(VI) (1000 ± 2 mg/L Cr, K_2_CrO_4_ in water), and Cr(III) (1000 ± 5 mg/L Cr in 2–3% HNO_3_) were purchased from Merck and used for the preparation of working standard solutions. SPS-SW1 Quality Control Material for Surface Water Analysis purchased from SPS Spectrapure Standards AS (Oslo, Norway) was used to check the accuracy of the determination of the total Cr concentrations in samples using ICP-MS, while Certified Reference Material Chromium Standard Solution 0.050 mg/L Cr(VI) ± 0.002 mg/L Cr(VI) K_2_CrO_4_ in H_2_O purchased from Merck, was used for verifying the accuracy of the determination of Cr(VI) with the HPLC–ICP–MS and spectrophotometric procedures. A WTW (Weilheim, Germany) 330 pH meter was employed to determine the pH of the samples.

**Figure 1:**
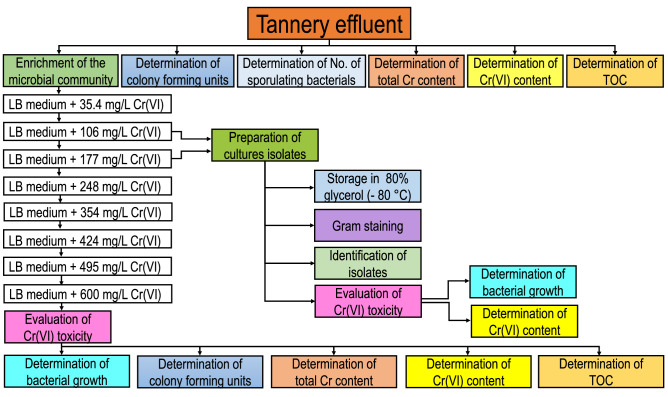
Flow chart showing the experimental set-up.

A flow chart showing the experimental set-up is presented in Fig. 1.

All the experiments were performed in three replicates.

## Results and discussion

### Accuracy check for the determination of the total Cr and Cr(VI) concentrations

The accuracy of the determination of total Cr concentrations using ICP-MS and Cr(VI) using the spectrophotometry and HPLC–ICP–MS procedures was verified by analyzing the SPS-SW1 reference material for the measurements of elements in surface waters, and CRM Chromium Standard Solution in H_2_O, respectively. The results, which are presented in Table S2, show that the determined values for Cr and Cr(VI) agreed well with the reported certified values. The differences did not exceed ± 2%, thereby confirming the accuracy of the analytical procedures applied.

The accuracy of the analytical procedure for the determination of Cr(VI) by spectrophotometry was also checked by the analysis of the LB medium treated with 500 mg/L Cr(VI) immediately and 48 h after the treatment using HPLC–ICP–MS and spectrophotometry. The results are presented in Table S3.

The data from Table S3 show good agreement of the results for the two speciation procedures in real samples. If not stated otherwise, due to its simplicity, high speed, low costs and large number of samples, spectrophotometry was further applied in the Cr(VI) analysis.

### Characteristics of the tannery effluent wastewater

The total Cr concentration in the tannery effluent wastewater determined using ICP-MS was 0.252 ± 0.007 mg/L, and the Cr(VI) determined using HPLC–ICP–MS was < 0.0005 mg/L. The TOC concentration determined by spectrophotometry was found to be 144 ± 7 mg/L, while the concentration of inorganic carbon was negligible. The pH of the sample was 7.1 ± 0.1. Since most of the environmental samples have neutral pH, Cr(VI) reduction using the isolates and microbial community was performed at pH 7.0 throughout the study. The effluent water was turbid and lightly yellow. The number of bacteria in the sample counted by colony forming units (CFU) on R2A agar plates was 1.4 × 10^3^ CFU/mL. No bacterial spores were detected, as no colonies were observed when the sample was first exposed for 20 min at 90 °C and then incubated on the LB agar plates for 24 h.

### Isolation of bacteria from enriched microbial communities treated with Cr(VI) and the identification of isolates

Several studies have shown that bacteria capable of growing at elevated concentrations of Cr(VI) and its reduction to trivalent Cr can be isolated from a variety of Cr-containing wastewaters. Among these, tannery effluents were frequently reported^26,30,33–35,37^. In the present work, wastewater effluent from the abandoned tannery was used. Ten morphologically different bacterial isolates were obtained from tannery effluent communities enriched in LB media with elevated concentrations of Cr(VI) according to the procedures described in section “Isolation and characterization of bacteria capable of growing at high concentrations of Cr”. Gram staining of the isolates showed their classification according to the type of cell walls (Gram positive (G^+^) or Gram negative (G^−^) bacteria) and their shape. To identify the strains, the partial 16S rRNA genes were amplified by PCR and sequenced (see section “Evaluation of Cr(VI) toxicity and its reduction”). Then the sequences were analyzed with MEGA 11 software and similarities with the existing strains in the database were found with the BlastN tool. The results are presented in Table 1.

**Table 1:** Characterization of the isolates.

Isolate	Concentration of Cr(VI) (mg/L) in enriched culture → Concentration of Cr(VI) (mg/L) in LB agar for isolation	Gram stain and shape	Species affiliation according to the partial 16S rRNA gene similarity	Similarity to the closest strain in the Genbank (%)	Identities
3001	106 → 106	G^−^ rod-shaped	*Pseudomonas aeruginosa*	100	1056/1056
3002	106 → 106	G^−^ rod-shaped	*Pseudomonas aeruginosa*	100	1062/1062
3003	106 → 106	G^−^ rod-shaped	*Pseudomonas aeruginosa*	100	1045/1045
3004	106 → 177	G^−^ rod-shaped	*Pseudomonas aeruginosa*	100	1037/1037
3005	106 → 177	G^+^ spherical	*Mammaliicoccus sciuri*	100	1188/1188
3006	106 → 177	G^−^ rod-shaped	*Pseudomonas aeruginosa*	100	1013/1013
3007	106 → 177	G^−^ rod-shaped	*Pseudomonas aeruginosa*	100	1018/1018
5008	177 → 177	G^−^ rod-shaped	*Pseudomonas aeruginosa*	100	1051/1051
5009	177 → 177	G^+^ spherical	*Mammaliicoccus sciuri*	100	1315/1315
50010	177 → 177	G^+^ spherical	*Mammaliicoccus sciuri*	100	1315/1315

The Gram staining data were consistent with the results of the isolates’ sequence analysis. Seven of the chromate-resistant bacterial strains belonged to the species *Pseudomonas aeruginosa *(*P. aeruginosa*), while three strains were affiliated with *Mammaliicoccus sciuri* (*M. sciuri,* formerly classified as *Staphylococcus sciuri*). *P*. *aeruginosa* is a rod-shaped G^−^ bacterium and was already shown to reduce Cr(VI)^17,26,42,43^*.* Due to its simple nutritional requirements and ability to adapt quickly to different environmental conditions, *P. aeruginosa* is frequently found in soil and water. *S. sciuri* are G^+^ spherical bacteria, found in soil and aquatic environments. Elahi and Rehman^34^ and Shahid et al.^44^ confirmed that *S. sciuri* can reduce Cr(VI).

### Influence of Cr(VI) toxicity on the bacterial growth and reduction capacity of the isolates

**Figure 2:**
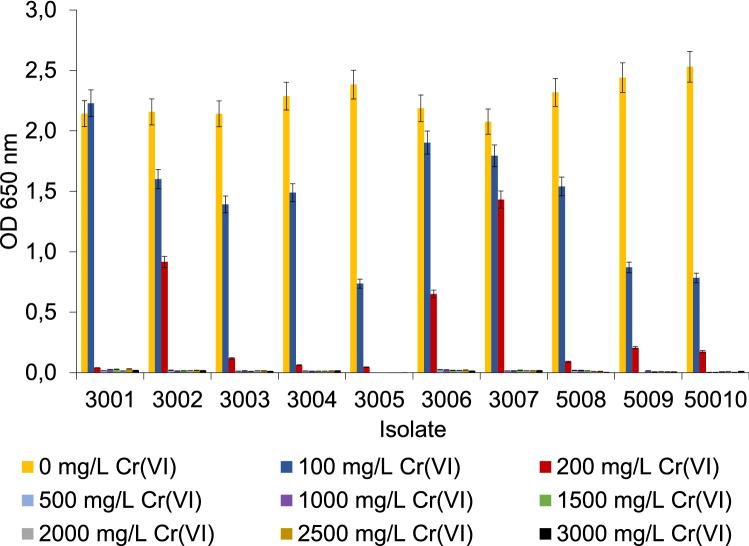
Optical cell density of individual isolates as a function of added Cr(VI) concentration to LB medium 24 h after incubation (48 h for isolate 5008).

To study the influence of Cr(VI) toxicity on the bacterial growth, the isolates were inoculated in the LB medium and treated with 0 to 3000 mg/L Cr(VI) (see procedure under section “Growth of bacterial isolates at various concentrations of Cr(VI) and its reduction effectiveness”). Growth of the isolates incubated for 24 h (48 h for the slow-growing isolate 5008) was monitored by measuring the optical cell density (OD_650_). The results are presented in Fig. 2.

The comparison of OD_650_ shown in Fig. 2 demonstrates that the *M. sciuri* isolates are more sensitive to the presence of Cr(VI). A concentration of 100 mg/L Cr(VI) inhibited the growth of the isolates 3005, 5009, and 50010, as the OD_650_ after 24 h is less than half of the OD_650_ when these strains were grown without added Cr(VI). In the presence of 100 mg/L Cr(VI), all the isolates affiliated with *P. aeruginosa* grow better than strains affiliated with *M. sciuri*. The concentration of 200 mg/L Cr(VI) in the medium strongly inhibits the growth of most isolates (3001, 3003, 3004, 3005, 5008, 5009, and 50010; OD_650_ value < 0.2); only three isolates, 3002, 3006, and 3007, of the isolates affiliated with *P. aeruginosa* reached OD_650_ values higher than 0.6. Concentrations of Cr(VI) above 500 mg/L are so toxic that they prevent any detectable growth of the studied isolates.

**Figure 3:**
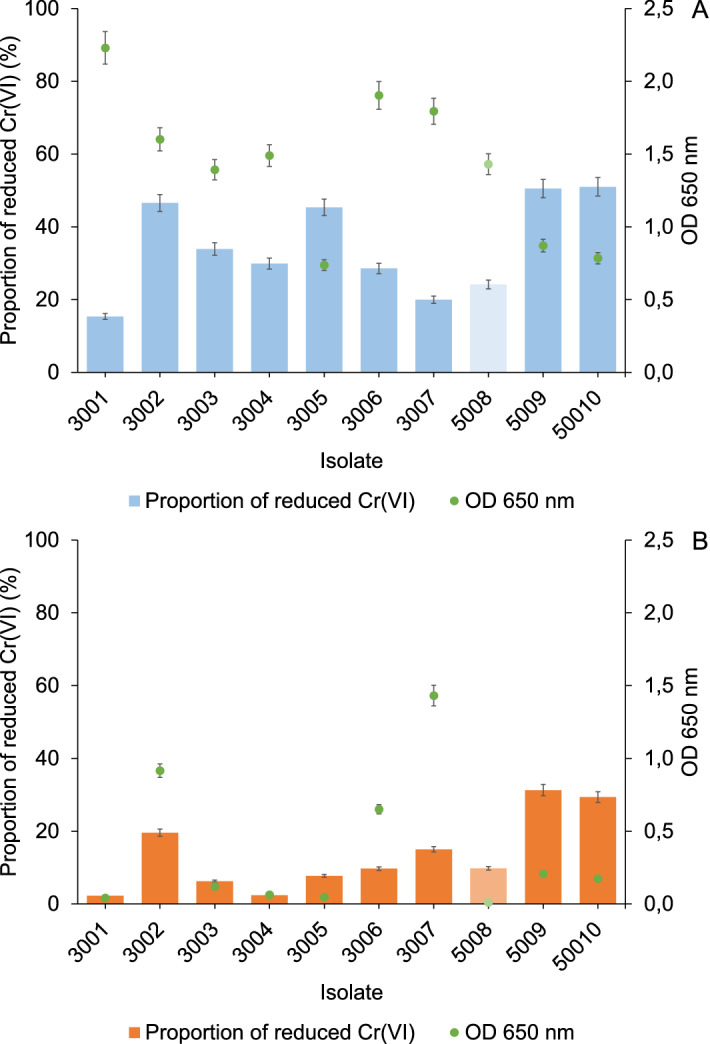
Proportion of reduced Cr(VI) and optical cell density of individual isolates as a function of added Cr(VI) concentration: (**A**) 100 mg/L and (**B**) 200 mg/L, to LB medium 24 h after incubation (48 h for isolate 5008).

To evaluate the bacterial reduction capacity, 100 or 200 mg/L of Cr(VI) were added to the isolates in the LB medium and samples were incubated for 24 or 48 h. After incubation, the bacterial cells were removed by centrifugation and filtering (0.45 µm) and Cr(VI) in the bacterial-free centrifugate was determined by spectrophotometry. The proportion of reduced Cr after 24 or 48 h was determined as the ratio between the measured and the added Cr(VI) concentration. The results are presented in Fig. 3.

The data in Fig. 3A show that the growth of the isolates and their reduction efficiency in the LB medium treated with 100 mg/L Cr(VI) differed significantly between the individual isolates. Although Cr(VI) inhibited the bacterial growth of the isolates affiliated with *M. sciuri*, the proportion of reduced Cr(VI) by these isolates (3005, 5009, and 50010) was higher (close to 50%) than the isolates affiliated with *P. aeruginosa* (isolates 3001, 3003, 3004, 3006, 3007, and 5008), which reached a higher OD_650_, but their reduction capacity was relatively low (15–30%). The only *P. aeruginosa* isolate with moderate growth and the ability to reduce about 50% of Cr(VI) in the tested conditions was isolate 3002.

Cr(VI) at 200 mg/L added to the LB medium (Fig. 3B) had a more toxic effect for the isolates studied (in general, very low OD_650_ values). Consequently, the bacterial reduction capacity was also low; in isolates 3001, 3003, 3004, 3005, and 5008, below 10%, while in isolates 3002, 5009, and 50010, between 20 and 30%. The results in Fig. 3 revealed that individual strains in the LB medium reduced different proportions of Cr(VI), whereby bacterial growth could be hindered by the toxic effect of Cr(VI). The greatest reduction capacity within the *P. aeruginosa* isolates was observed for isolate 3002, which reduced about 50% of added Cr(VI) with a concentration of 100 mg/L, and 20%, with a concentration of 200 mg/L.

Li et al.^45^ investigated the bioreduction of Cr(VI) on goethite (FeOOH) in the presence of *P. aeruginosa* isolate (AB93066) under aerobic conditions. The bacterium was obtained from China’s national collection of microorganisms at Wuhan University. At pH 5.5, 45 °C and an incubation time of 60 h, 20 mg/L Cr(VI) was completely removed, while for effective removal of 35 mg/L Cr(VI), a longer incubation time was necessary (100 h). These results are comparable to our findings, as *P. aeruginosa* strains isolated from tannery effluents were in general able to reduce 20 to 50% of the Cr(VI) at its initial concentration of 100 mg/L (pH 7, 37 °C, 24 h). Other Cr(VI)-tolerant bacteria from *Bacillus* species (*B. amyloliquefaciens*, strain CSB 9), isolated from chromite mine soil, have shown the ability to completely reduce 100 mg/L of Cr(VI) within 144 h^31^, while *Lactobacillus* strains were able to entirely reduce 32 mg/L Cr(VI) within 6 h^27^. Tan et al.^46^ reported that the *Bacillus *sp*.* CRB-B1 strain, isolated from sewage sludge, could reduce 97% of an initial Cr(VI) concentration 150 mg/L (pH 7, 37 °C, 24 h), using glucose and fructose as the electron donors, which enhanced the reduction capacity. Elahi and Rehman^34^ reported that bacterium *S. sciuri* (A-HS1), isolated from tannery effluent, was able to remove 93% of Cr(VI) containing 104 mg/L Cr(VI) within 6 days of incubation at 37 °C and pH 7. In our investigation, three isolates of *S. sciuri* (3005, 5009, 50010) were able to reduce 50% of Cr(VI) with an initial concentration of 100 mg/L, but we applied a shorter incubation time (24 h). Among the other bacteria present in the tannery effluents, Bharagava and Mishra^47^ isolated the bacterium *Cellulosimicrobium *sp*.* (KX710177), which can completely reduce 50 mg/L Cr(VI) in an incubation time of 24 h. For the effective reduction of 100 mg/L Cr(VI), a longer incubation time of 96 h was necessary. Singh et al.^33^ isolated a chromate-resistant facultative anaerobic bacterial strain (FA-3) of *B. cereus* species from a treated tannery effluent, which showed the ability to reduce 72% of the Cr(VI) at an initial concentration of 1000 mg/L over a wide range of pH and incubation temperatures (pH 6–10, 25–40 °C, 90 h). However, in our experiments, at Cr(VI) concentrations above 500 mg/L, the bacterial growth was completely inhibited (Fig. 2). Other researchers also observed the toxic effects of Cr(VI), which hindered the bacterial growth in concentrations higher than 32 mg/L Cr(VI)^27,45^, 100 mg/L (Cr(VI) (Das et al.^31^) and 300 mg/L Cr(VI)^46,47^.

### Influence of Cr(VI) toxicity on the growth of the enriched microbial community and its reduction capacity

**Figure 4:**
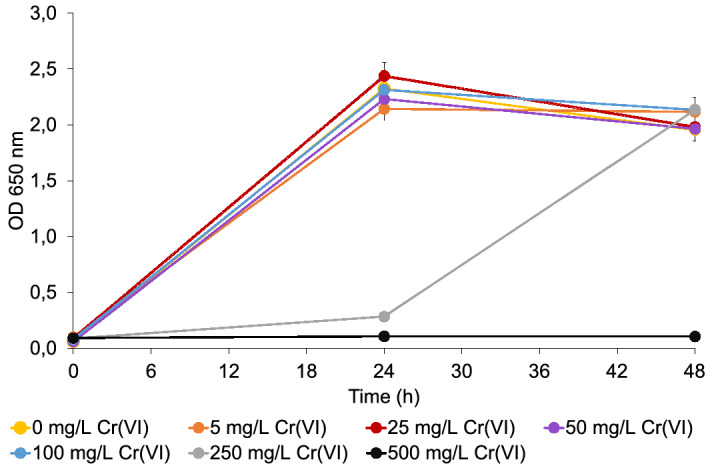
Influence of different Cr(VI) concentrations on cell density (OD at 650 nm) of enriched microbial community after 24 and 48 h of incubation in LB media.

To evaluate the influence of Cr(VI) toxicity on the growth, an enriched microbial community treated with 600 mg/L Cr(VI) was used. The concentration of Cr(VI) in the inoculum was below 0.01 mg/L. The cells were re-suspended in the LB medium treated with 5, 25, 50, 100, 250 or 500 mg/L Cr(VI), as described in section “Molecular identification of the isolates”, and the bacterial growth was examined with optical cell density at time zero, 24, or 48 h after incubation. The same experiment was performed without the addition of the inoculum to media treated with Cr(VI). The results are presented in Fig. 4.

The data in Fig. 4 demonstrate that the growth of the bacteria depended on the incubation time and on the Cr(VI) concentration in the LB medium. Concentrations of added Cr(VI) up to 100 mg/L did not inhibit the growth of the bacterial community, as 24 h after incubation the OD_650_ was similar to that in the LB medium without the addition of Cr(VI). 250 mg/L of Cr(VI) in the LB medium had an inhibitory effect on the growth of the microbial community. In the first 24 h after incubation, very low OD_650_ values were observed. However, with longer incubation times the microorganisms adapted to the presence of high Cr(VI) concentrations. Within the next 24 h the bacteria began to grow, and after 48 h the OD_650_ values approached the values of the untreated community. At very high concentrations of Cr(VI), i.e., 500 mg/L, bacterial growth was severely inhibited and also hindered by a prolonged incubation time. The OD_650_ value did not increase, even after 48 h, indicating a high Cr(VI) toxicity. The data in Fig. 4 further show that bacterial growth in the presence of up to 100 mg/L Cr(VI) in the microbial community is better than the growth of the individual isolates (see Fig. 2). This means that the bacteria in the microbial community adapt better to elevated concentrations of toxic Cr(VI) than the individual strains.

**Figure 5:**
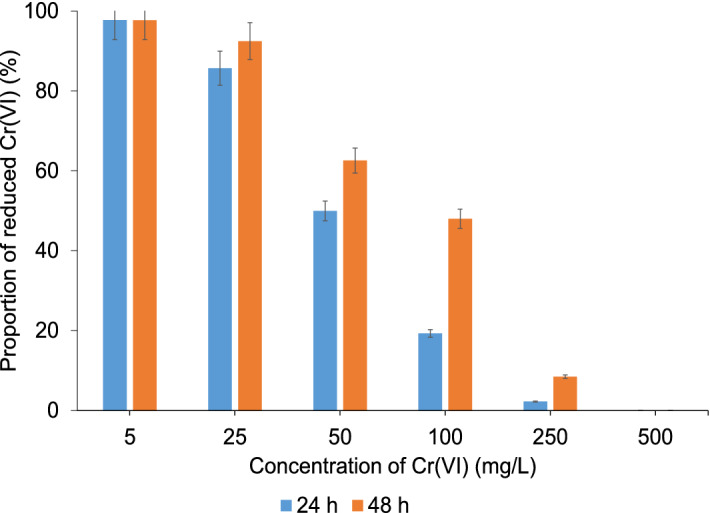
Proportion of reduced Cr(VI) by enriched microbial community in LB media treated with different Cr(VI) concentrations 24 or 48 h after incubation.

For assessing the microbial reduction capacity, Cr(VI) was determined in the LB media with the addition of the enriched microbial community treated with Cr(VI) concentrations from 5 to 500 mg/L, 24 or 48 h after incubation. The results are presented in Fig. 5.

The data from Fig. 5 indicate that the enriched microbial community added to the LB medium was able to completely reduce the Cr(VI) at concentrations up to 5 mg/L within 24 h. At higher Cr(VI) concentrations, the rate of Cr(VI) reduction increased with time, whereas the reduction efficiency of the bacteria decreased with an increasing Cr(VI) concentration. At a concentration of 100 mg/L, the bacteria reduced 50% of the Cr(VI) within 48 h, while they were ineffective at reducing the 500 mg/L Cr(VI). The latter concentration of Cr(VI) is toxic to bacteria, which is also evident from the negligible cell density in the LB medium (Fig. 4). A comparison of the data from Figs. 3 and 5 also showed that the bacteria from the enriched microbial community can reduce the same amount of Cr(VI) in 48 h as the best individual isolates of strains affiliated with *M. sciuri* and *P. aeruginosa* (isolate 3002) in 24 h.

Although individual isolates might be more successful in reducing Cr, the community of different bacterial species and strains adapts more easily to stressful environmental conditions, and thus develops more mechanisms of resistance to Cr(VI).

**Figure 6:**
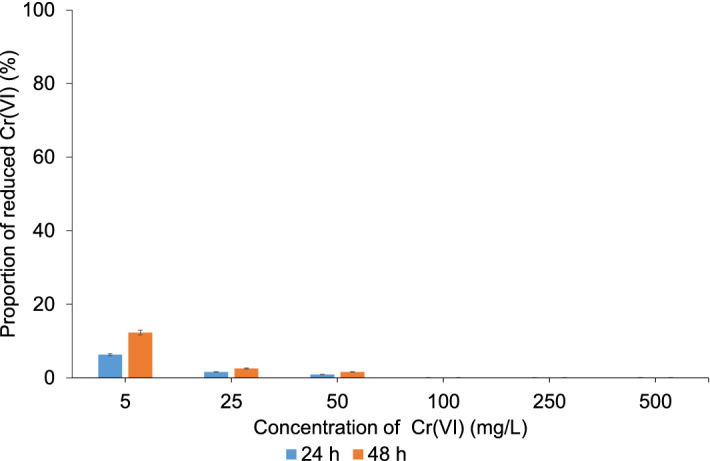
Proportion of reduced Cr(VI) in LB media treated with different Cr(VI) concentrations 24 or 48 h after incubation.

To distinguish between the proportion of Cr(VI) reduced by the bacteria and that reduced by the organic matter and/or other reducing agents present in the LB medium (e.g., Fe(II), sulfides, sulfites), the same experiment was performed as with the enriched microbial community, except that no inoculum was added to the LB media treated with Cr(VI). The results are shown in Fig. 6.

The concentration of organic carbon in the LB medium, determined using spectrophotometry, was 897 ± 45 mg/L. The data in Fig. 6 revealed that despite the relatively high content of total organic carbon, the reduction efficiency of Cr(VI) with organic matter and other reducing agents was low. After 48 h, only 13% of the Cr(VI) with a concentration of 5 mg/L was reduced, while at Cr(VI) concentrations of 25 and 50 mg/L, the reduction efficiency was only around 3% and was negligible at higher Cr(VI) concentrations. These data confirmed that in the LB medium with an enriched microbial community, the reduction takes place predominantly with microorganisms.

Ma et al.^36^ also investigated the ability of a mixed bacterial consortium obtained from a chromium-contaminated soil for the remediation of groundwater contaminated with Cr(VI). The bacterial consortium was cultivated in an acetate medium containing a mixture of salts, yeast extract, and K_2_HPO_4_ as a buffer. At pH 8, 30 °C and a bacterial inoculum of 10% (v/v), 20 mg/L Cr(VI) was completely reduced by the bacteria within 5 days. The enriched microbial community in this investigation showed even greater potential for Cr(VI) reduction.

## Conclusions

The microbial community from tannery effluent was enriched to be able to grow at elevated Cr(VI) concentrations. Seven chromate-resistant bacterial strains affiliated with *P. aeruginosa* and three with *M. sciuri* were isolated and identified by 16S rRNA gene sequence analyses. Increasing Cr(VI) concentrations in the LB medium inhibited the growth of bacteria, but the amount of bacteria did not necessarily correlate with the amount of Cr(VI) reduced. Isolates affiliated with *M. sciuri* were more sensitive to the presence of Cr(VI) than isolates affiliated with *P. aeruginosa*, but their reduction capacity was higher than that of *P. aeruginosa* isolates. About 50% of the Cr(VI) with an initial concentration of 100 mg/L Cr(VI) was reduced by *M. sciuri* strains (3005, 5009, 50010) within 24 h of incubation, while only one of the seven *P. aeruginosa* isolates (3002) grew well and also reduced around 50% of the Cr(VI). Bacterial growth in the presence of up to 100 mg/L Cr(VI) in the enriched microbial community was better than the growth of most individual isolates, as bacteria in the microbial community adapted better to elevated Cr(VI) concentrations than in individual strains. The reduction capacity of the bacteria in the microbial community was similar to that of the best individual isolated strains, but the community needed a longer incubation time (48 h) to achieve a comparable reduction of Cr(VI). Compared to the active biological Cr(VI) reduction by bacteria, the chemical reduction with organic matter in the LB medium was negligible. The effective, safe and rapid reduction of toxic Cr(VI) by the isolated strains or by the enriched microbial community at environmentally relevant concentrations that can be found in the Cr(VI) polluted sites suggests the possibility of establishing a system for the continuous removal of Cr(VI) from industrial effluents.

## Supplementary Information

Supplementary Tables.

## Data Availability

The datasets generated and/or analyzed during the current study are available in the [GenBank database under accession numbers ON409639-ON409641 and ON430687-ON430693, NCBI database (National Center for Biotechnology Information)] repository, [PERSISTENT ACCESSION NUMBER TO DATASETS ON409639, ON409640, ON409641, ON430687, ON430688, ON430689, ON430690, ON430691, ON430692, ON430693].
