# Supplementary material for: Reduction of hexavalent chromium using bacterial isolates and a microbial community enriched from tannery effluent
Source: Sci Rep. 2022 Nov 23;12:20197. doi: 10.1038/s41598-022-24797-z (PMC9684402; doi:10.1038/s41598-022-24797-z)
Supplement: Supplementary file 1 — Supplementary Tables. [file 41598_2022_24797_MOESM1_ESM.docx]

**Table S1**

ICP-MS operating parameters.

| Parameter | Type/Value  Speciation analysis | Type/Value  Total element concentration analysis |
| --- | --- | --- |
| *Sample introduction* |  |  |
| Nebuliser | Miramist | Miramist |
| Spray chamber | Scott | Scott |
| Skimmer and sampler | Ni | Ni |
| *Plasma conditions* |  |  |
| Forward power | 1550 W | 1550 W |
| Plasma gas flow | 15.0 L/min | 15.0 L/min |
| Carrier gas flow | 0.75 L/min | 1.05 L/min |
| Dilution gas flow | 0.45 L/min | 0.10 L/min |
| He gas flow | 10 mL/min | 4.5 mL/min |
| QP bias | -97 V | -15 V |
| Oct bias | -100 V | -18 V |
| Cell entrance | -130 V | -40 V |
| Cell exit | -150 V | -60 V |
| Deflect | -80 V | -2.2 V |
| Plate bias | -150 V | -60 V |
| Sample uptake rate | 1.5 mL/min | 0.3 mL/min |
| *Data acquisition parameters* |  |  |
| *m/z* of isotopes monitored | ^52^Cr | ^52^Cr |
| *m/z* of internal standard | ^103^Rh | ^103^Rh |
| Total acquisition time | 600 s |  |

**Table S2**

Total Cr concentrations in SPS-WW1 determined by ICP-MS, and Cr(VI) in CRM Chromium Standard Solution in H_2_O determined by spectrophotometry and HPLC-ICP-MS. The results represent the average of three parallel determinations.

| Parameter | SPS-SW1 Cr_tot_  ICP-MS | | CRM Cr(VI) in H_2_O  Spectrophotometry | | CRM Cr(VI) in H_2_O  HPLC-ICP-MS | |
| --- | --- | --- | --- | --- | --- | --- |
|  | Deteremined  (ng/mL) | Certified  (ng/mL) | Deteremined  (ng/mL) | Certified  (ng/mL) | Deteremined  (ng/mL) | Certified  (ng/mL) |
| Total Cr | 1.99 ± 0.03 | 2.00 ± 0.02 | / | / | / | / |
| Cr(VI) | / | / | 48.1 ± 1.7 | 47 ± 2 | 46.0 ± 1.0 | 47 ± 2 |

**Table S3**

Concentrations of Cr(VI) in LB medium treated with 500 mg/L Cr(VI) determined at t_0_ and t_48_ using spectrophotometry and HPLC-ICP-MS. Results represent the average ± standard deviation of three parallel samples.

| LB medium +  500 mg/L Cr(VI) | Spectrophotometry  Cr(VI) (mg/L) | HPLC-ICP-MS  Cr(VI) (mg/L) |
| --- | --- | --- |
| t_0_ | 480 ± 15 | 470 ± 10 |
| t_48_ | 460 ± 15 | 450 ± 10 |
